# Lack of local genetic representation in one of the regions with the highest bird species richness, the Peruvian Amazonia

**DOI:** 10.1371/journal.pone.0296305

**Published:** 2024-01-02

**Authors:** Alejandra Arana, César Arana, Mrinalini Watsa, Mathias W. Tobler, Víctor Pacheco, Juan Esteves, José Luis Mena, Letty Salinas, Jorge L. Ramirez

**Affiliations:** 1 Museo de Historia Natural, Universidad Nacional Mayor de San Marcos, Lima, Peru; 2 Facultad de Ciencias Biológicas, Universidad Nacional Mayor de San Marcos, Lima, Peru; 3 San Diego Zoo Wildlife Alliance, Conservation Science and Wildlife Health, Escondido, California, United States of America; 4 San Diego Zoo Wildlife Alliance Peru, Cusco, Peru; Instituto Leonidas e Maria Deane Fiocruz Amazonia, BRAZIL

## Abstract

Peru ranks among the three countries with the highest bird species diversity globally and a majority of those species are found in the Peruvian Amazon. However, birds in this area are currently facing serious anthropogenic threats. Genetic and genomic methods are becoming important tools for avian biodiversity monitoring and conservation planning. Comprehensive molecular libraries that are publicly available are key to the effective deployment of these tools. We analyze the information gaps for four molecular markers in the most important genetic sequence databases, Barcode of Life Data Systems (BOLD) and NCBI GenBank, for bird species of the Peruvian Amazonia. We found that 64% of Peruvian Amazonian bird species have gene sequences for COI, 59.5% have CYTB sequences, 16.4% have 12S sequences, and only 0.6% have 18S sequences. However, these numbers decrease drastically to 4.3% for COI sequences when we only consider specimens sampled in Peru. Our data also showed that 43.8% of Peruvian Amazonian endemic species (n = 32) are missing sequences of any screened marker uploaded to GenBank or BOLD. Our results will encourage and guide efforts of the scientific community to complete reference libraries for Peruvian avian species that will be useful for future DNA-based monitoring projects that include birds.

## Introduction

The Amazon rainforest is a mega-diverse region [1] where studies have been conducted to understand speciation processes [2], habitat specialization [3], and reproduction strategies [4] among other core biological and ecological questions. One of the most diverse and well studied animal groups in the Amazon rainforest are birds [5]. Peru ranks among the most biodiverse countries in terms of bird diversity, harboring around 17% of global bird species [6, 7]. Exploration in the Peruvian Amazon rainforest has had a great focus on birdlife since early European and North American expeditions in the 1800s [8] and has provided a long-term record of bird diversity in the region. However, Peruvian avifauna is currently facing several threats such as habitat loss and modification due to agricultural expansion (particularly oil palm production [9]), illegal gold mining [10], poaching [11], and illegal wildlife trade [12].

Today, DNA-based approaches for species identification, such as DNA barcoding and eDNA metabarcoding, are helping to facilitate comprehensive biodiversity monitoring programs and inform conservation measures [13–17]. However, these methodologies rely on publicly available molecular reference libraries for accurate species identification [13]. Furthermore, a comprehensive genetic reference library is crucial to promote Peruvian scientific research to comply with the Convention on Biological Diversity 2030 Targets, specifically target 4 which highlights the urgency for management measures for the recovery and conservation of species, in particular endangered species, and for restoring their genetic diversity (https://www.cbd.int/gbf/targets).

Although several research groups worldwide continue to work on near-universal and relatively low-cost protocols to improve reference libraries, mainly within the framework of DNA barcoding [18, 19], previous studies of information gaps in the region show that only 22.7% of birds from Loreto (the largest Amazonian department in Peru) have sequences deposited in a public molecular database [20].

Mitochondrial markers are common targets in eDNA and metabarcoding projects due to the high number of copies of them that can be in each cell and due to their high availability in reference databases [21, 22]. Among the most widely used molecular markers for metabarcoding projects in vertebrates, Cytochrome C Oxidase subunit I (COI) has become the most popular thanks to worldwide DNA barcoding programs [23]. Cytochrome b (CYTB) is widely used as a marker for phylogenetic and phylogeographic studies of birds, including studies focused on calibrating the molecular clock in birds [24]. 12S and 18S are also among the most common environmental eDNA metabarcoding markers for wildlife monitoring research [25] and provide more conserved priming sites that can increase amplification success compared to COI or CYTB, but they have lower species identification success [26]. Here, we present an updated analysis of the sequence information for avian species from the Peruvian Amazonia contained in two of the most important genetic sequence databases, Barcode of Life Data Systems (BOLD) and NCBI GenBank, focusing on four commonly used molecular markers: Cytochrome C Oxidase I (COI), Cytochrome b (CYTB), 12S rDNA (12S) and 18S rDNA (18S).

## Materials and methods

### Peruvian Amazonian bird species list

We compiled potential lists of all Peruvian Amazonian birds using data from Avibase (www.avibase.org) and the revised species list of the Loreto region [27], organizing this information according to the International Ornithological Committee (IOC) classification system [6]. In this study, we define Amazonian species as those that occur in forested habitats on the eastern slope of the Andes, in Amazon montane and lowland forests, which correspond to the Yunga region and tropical rainforest in the National Map of Ecosystems [28]. These forests occur from sea level to 3500 meters of altitude [28]. In addition to our literature search we incorporated recent curated records from the citizen science platform eBird (ebird.org).

We screened the following departments to build our Peruvian Amazonian bird species list: Madre de Dios, Ucayali, Loreto, Amazonas, San Martín, Huánuco, Pasco, Junín, Ayacucho, Cusco, and Puno. We performed an exhaustive search of the distribution of each species, to ensure that only Peruvian Amazonian species were considered.

### Identification of information gaps in public molecular libraries (BOLDsystems and GenBank)

We performed a search for information gaps for four molecular markers commonly used in phylogeny, biogeography, ecology, and environmental DNA analysis, among others: Cytochrome C Oxidase I (COI), Cytochrome b (CYTB), 12S rDNA (12S) and 18S rDNA (18S). These markers are sequenced in various projects using different primers and obtaining a variety of fragment sizes, from full genes down to mini barcodes of ≤100 base pairs [14, 16]. Therefore, we did not include a minimum length limit in our search. We compared our curated list of birds of Peru with the list of species sequenced in Barcode of Life Data system (BOLD) [29] using the ’bold_seqspec’ function of the ’bold’ package in R [30] and the BOLD public API (v3.boldsystems.org/index.php/api_home). We used the R package "Rentrez" for the sequence search in GenBank [31].

Considering that GenBank and BOLD do not regularly update taxonomic name changes according to IOC system, we also searched for synonymous and the most recent names available in Avibase (www.avibase.org). Geographic coordinates for sample collection sites were only available for sequences uploaded to BOLD. Conservation status allocations were based on International Union for Conservation of Nature Red List (IUCN) [32], which classifies a species as threatened if it has been assessed as Critically Endangered (CR), Endangered (EN), or Vulnerable (VU).

We used ArcGIS 10.3 to evaluate which samples originated from Peru. Additionally, we used shapefiles from University of Copenhagen Center for Macroecology, Evolution and Climate (https://macroecology.ku.dk/resources/wallace) [33], the Oak Ridge National Laboratory Distributed Active Archive [34], and the tool Intersect from Analysis Tools in ArcGIS 10.3 to determine if the sampling points were located inside the same zoogeographic realm [33] as the Peruvian Amazonia (the Neotropical realm) and inside the Amazon Basin [34].

We conducted a DNA barcode quality analysis based on Oliveira et al. [35]. This analysis is based on the Barcode Index Number (BIN), which is a molecular approach to delimit Molecular Operational Taxonomic Units (MOTUs) [36]. BINs are an effective method for identifying the possible existence of cryptic species and hidden diversity [36].

This barcode quality analysis method species get “A/B” when a species has ≥3 barcodes and their specimens are clustered in the same BIN (A if the barcodes come from independent studies, and B if all sequences come from the same dataset); “C” when a species has ≥3 barcodes, and their specimens are clustered in different BINs and those BINs only contain 1 species (i.e. target species); “D” when a species has <3 barcodes (i.e. is data deficient); “E*” when a species has ≥3 barcodes, their specimens are clustered in the same BIN and that BIN contains ≥1 species; “E**” when a species has ≥3 barcodes, their specimens are clustered in different BINs and those BINs contain ≥1 species; and “F” when all the specimens of a species are either unvouchered or directly mined from GenBank (i.e. inadequate DNA barcoding procedure) [35]. We used the AuditionBarcodes function in the BOLDmineR package (github.com/ulises-rosas/boldminer) for this analysis.

## Results

### Database of Peruvian Amazonian bird species

Our data indicated that out of the 1894 bird species present in Peru [7], 1506 species (79.5%) can be considered as Amazonian bird species (S1 File). Likewise, out of the 110 endemic Peruvian bird species [7], 73 species have been recorded in Amazonian regions (S1 File).

### Identification of information gaps

**Fig 1:**
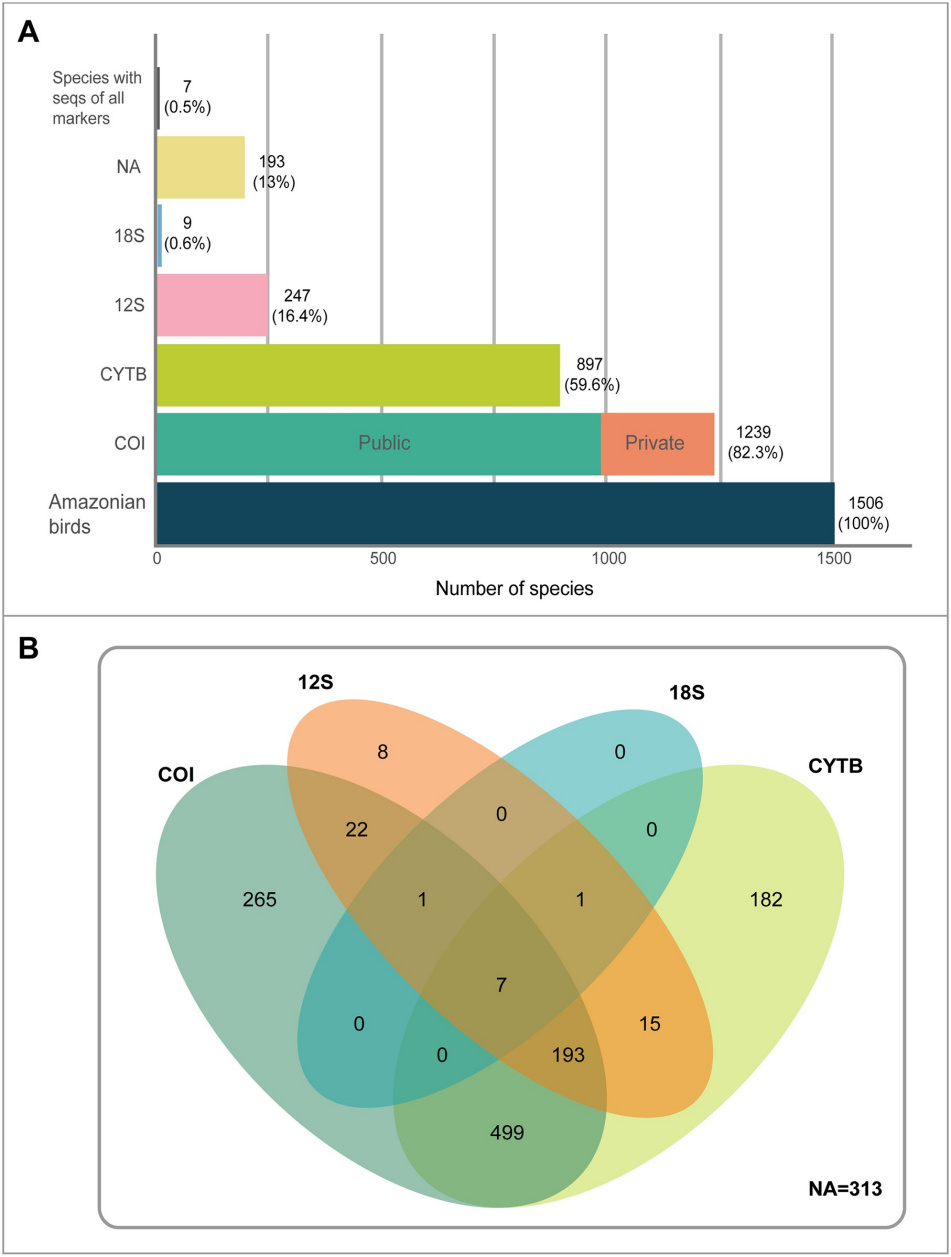
A) Number of Peruvian Amazonian species represented by COI, CYTB, 12S or 18S sequences uploaded to online molecular libraries (BOLD and GenBank). B) Venn diagram of number of species registered with each genetic marker in public molecular libraries (BOLD and GenBank). Values in intersections indicate the number species that have molecular information for two or more markers. NA = bird species with no public records in BOLD or GenBank.

We found that 897 (59.6%) of avian species of the Peruvian Amazon have CYTB sequences in GenBank, 247 species (16.4%) have 12S sequences, and only 9 species (0.6%) have 18S sequences (Fig 1A). COI was the marker with the largest number of species represented in public reference libraries, with 1239 species (82.3%) registered in GenBank and BOLD. However, 252 of these are private, and therefore only 987 species (65.5%) have COI sequences available to the public (S1 File, S1 Table).

For Peruvian Amazonian endemic species, 43.8% (32 of 73 species) do not have sequences for the analyzed markers uploaded to GenBank or BOLD. Furthermore, only 7 species have sequences for all four markers (Fig 1B).

There is a high level of variation among the length of the sequences of the markers analyzed. The shortest sequences had a length of 113 base pairs. On the other hand, most CYTB sequences had lengths of 1000 base pairs, while COI sequences most frequent lengths have between 644 and 696 base pairs.

**Fig 2:**
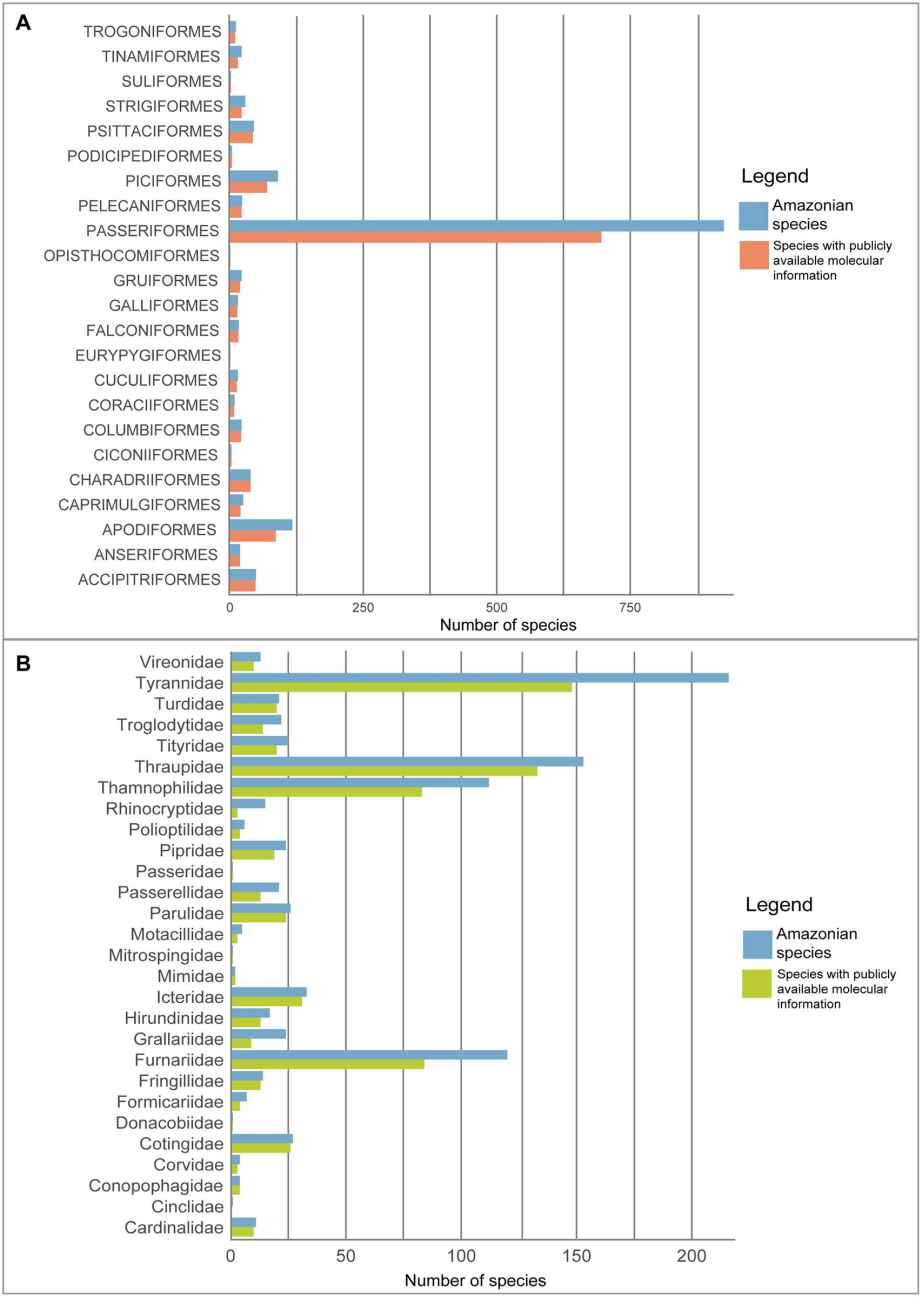
A) Number of Peruvian Amazonian bird species of each order that are represented by public sequences of at least one of the four markers analyzed in GenBank or BOLD. B) Number of Peruvian Amazonian bird species of each family of the Passeriformes order that are represented by public sequences of at least one of the four markers analyzed in GenBank or BOLD.

The bird orders with the highest number of Amazonian bird species represented with these markers in BOLD and GenBank were the Passeriformes (791 species, 85.4% of the total representation), followed by Apodiformes (92 species, 78.6%), and Piciformes (80 species, 88.9%), while Accipitriformes, Charadriiformes and Psittaciformes had more than 30 species each (S1 Table, Fig 2A). The families with the highest number of species represented with public data were Tyrannidae (148 species, 68.5%), Thraupidae (133 species, 86.9%), Furnariidae (84 species, 70%), Thamnophilidae (84 species, 75%), and Trochilidae (77 species, 74%) (Fig 2B).

**Fig 3:**
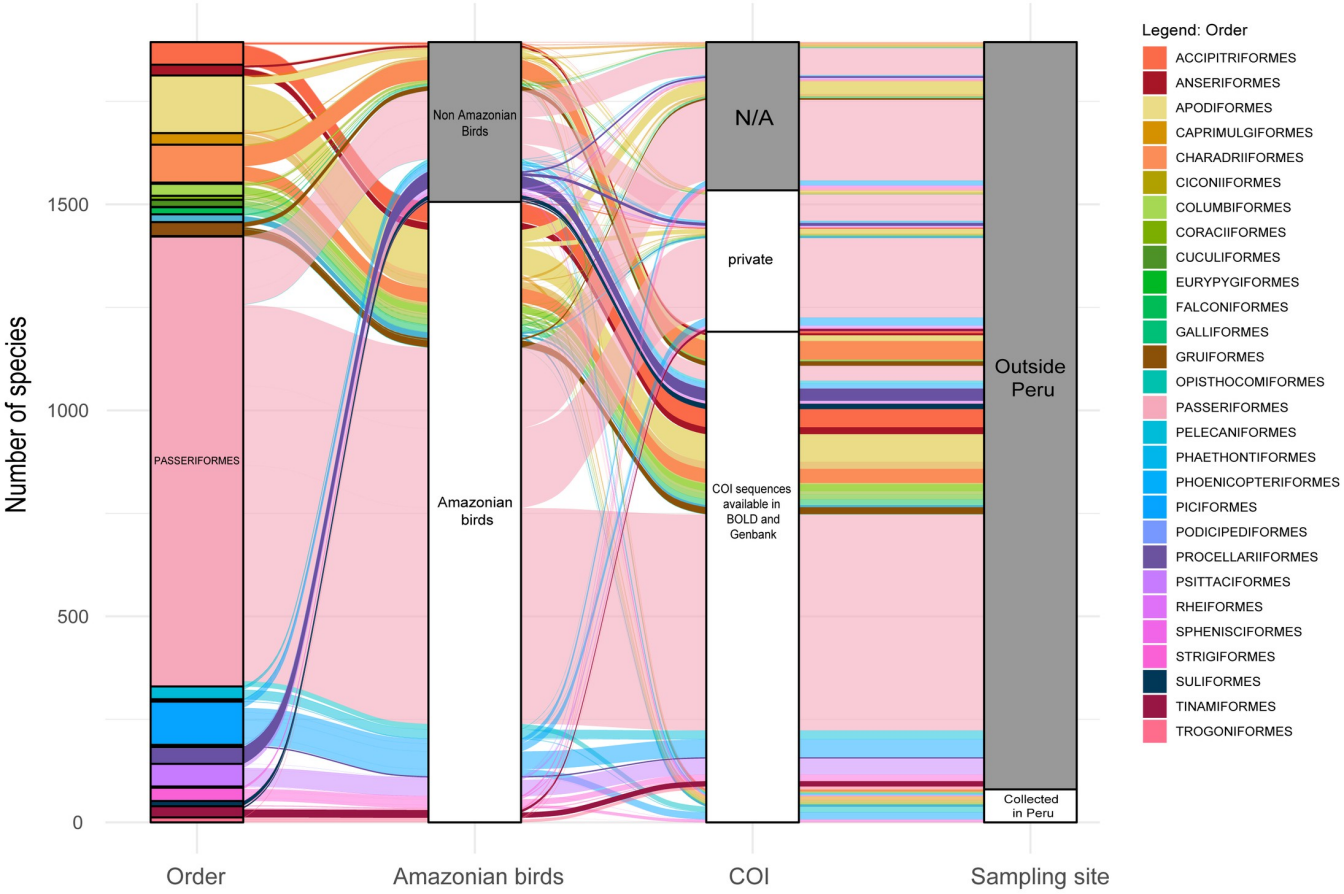
Number of Peruvian Amazonian bird species represented by COI sequences in BOLD, indicating the percentage of how many of those come from samples collected in Peru. N/A = bird species with no records in BOLD or GenBank.

**Fig 4:**
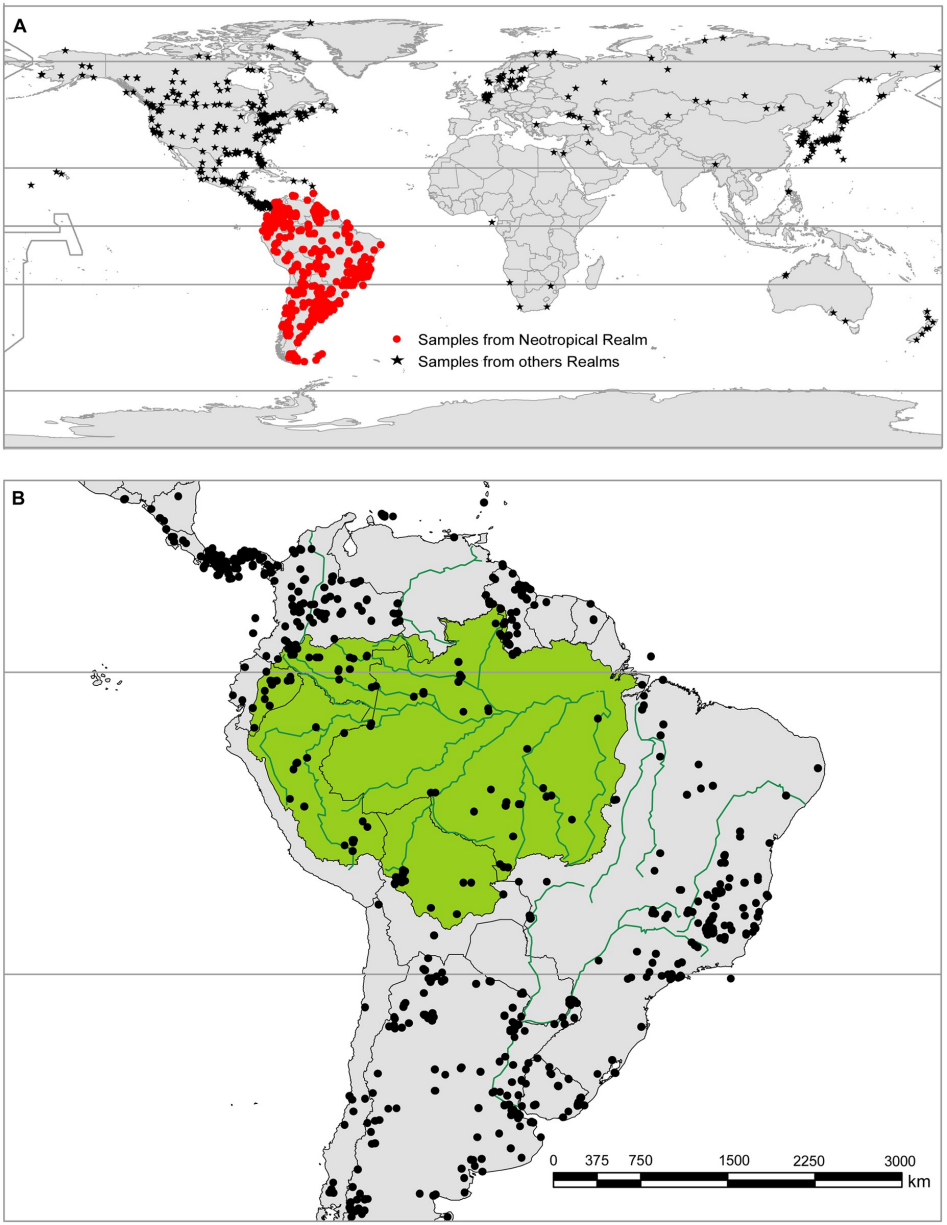
Localities registered in BOLD where the sequences of bird species analyzed in this study come from. Here, we show the global geographic distribution of all sequenced samples that represent bird species reported in the Peruvian Amazonia. A) A significant number of these sequences were obtained from samples taken from outside the Neotropical zoogeographic realm, as indicated by black stars. Red dots indicate samples from within the Neotropical realm [33]. B) The map shows the Amazon Basin in green, and rivers in turquoise. It shows the number of sample localities (black dots) outside of the Amazon Basin. Shapefiles of the Amazon River were obtained from the Oak Ridge Natioanal Laboratory Distributed Active Archive CEnter, which is under public domain [34]. Grey horizontal lines delimit the tropics. Free vector data from Natural Earth (https://www.naturalearthdata.com/), which is under public domain.

However, our data show that only a small subset (n = 64) of the sequences found on BOLD system come from samples collected in the Peruvian Amazonia (Fig 3). Furthermore, of the georeferenced samples in BOLD, while 92% of Peruvian Amazonian bird species are represented by at least one sequence taken from the Neotropical realm, only 68% of the total number of sequences comes from samples collected in this realm (Fig 4A). Similarly, 59% of species were represented by samples from outside the Amazon Basin, and 82.8% of sequences correspond to sampling localities outside this basin (Fig 4B).

**Fig 5:**
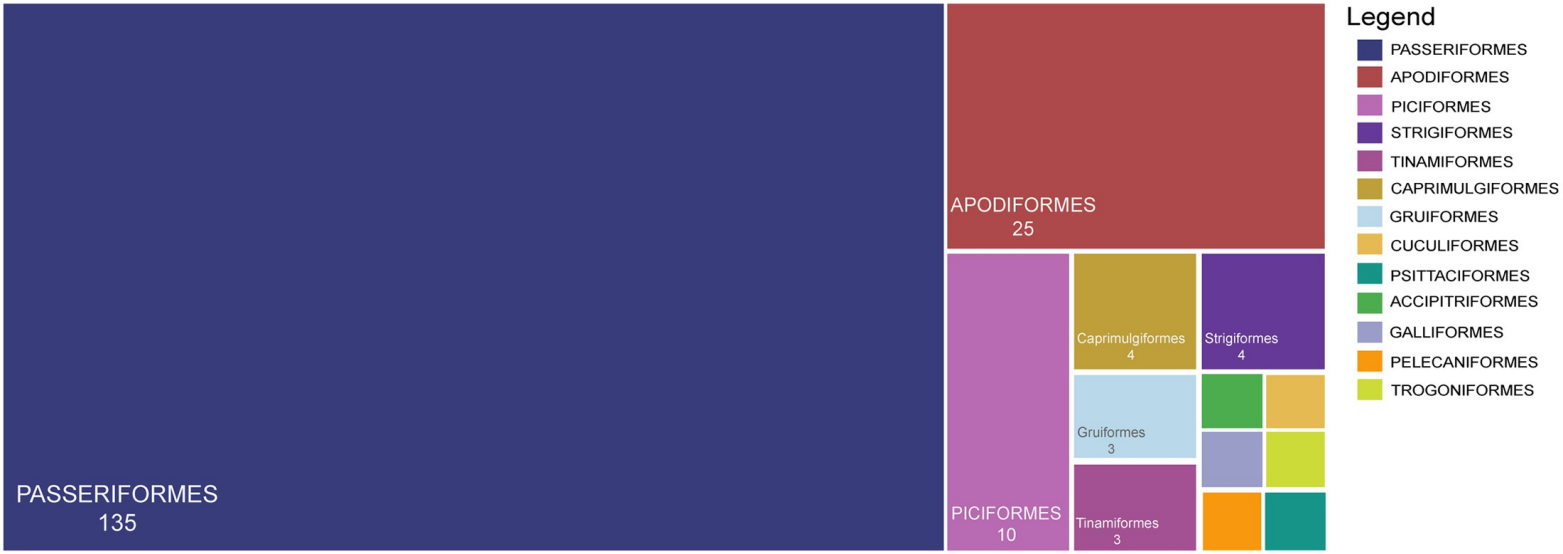
Number of Peruvian Amazonian bird species per taxonomic order that do not have sequences for any of the four molecular markers analyzed.

We found 190 Peruvian Amazonian bird species that have no sequences for any of the four markers analyzed. Most of these belong to the order Passeriformes (135 species), Apodiformes (25 species), and Piciformes (10 species) (Fig 5). There are 14 species of Peruvian Amazonian birds that are threatened according to the IUCN and do not have sequences for COI, CYTB, 12S or 18S, half of which are also endemic Peruvian species (Table 1).

**Table 1:** List of threatened Peruvian Amazonian bird species that have no information of the four markers analyzed.

Family	Scientific name	English name	Conservation status
Cracidae	*Pauxi koepckeae* (E)	Sira Curassow	CR
Trochilidae	*Loddigesia mirabilis* (E)	Marvelous Spatuletail	EN
Ramphastidae	*Aulacorhynchus huallagae* (E)	Yellow-browed Toucanet	EN
Thamnophilidae	*Euchrepomis sharpei*	Yellow-rumped Antwren	EN
Thamnophilidae	*Myrmoborus lugubris*	Ash-breasted Antbird	VU
Thamnophilidae	*Myrmoborus melanurus*	Black-tailed Antbird	VU
Grallariidae	*Grallaricula ochraceifrons* (E)	Ochre-fronted Antpitta	VU
Furnariidae	*Synallaxis courseni* (E)	Apurimac Spinetail	VU
Tyrannidae	*Cnipodectes superrufus*	Rufous Twistwing	VU
Tyrannidae	*Poecilotriccus luluae* (E)	Johnson’s Tody-Tyrant	EN
Tyrannidae	*Zimmerius cinereicapilla*	Red-billed Tyrannulet	VU
Tyrannidae	*Phyllomyias weedeni*	Yungas Tyrannulet	VU
Tyrannidae	*Agriornis albicauda*	White-tailed Shrike-Tyrant	VU
Thraupidae	*Cnemathraupis aureodorsalis* (E)	Golden-backed Mountain-Tanager	EN

Endemic species have (E) next to their scientific name. Conservation status is based on IUCN Red List [32].

**Fig 6:**
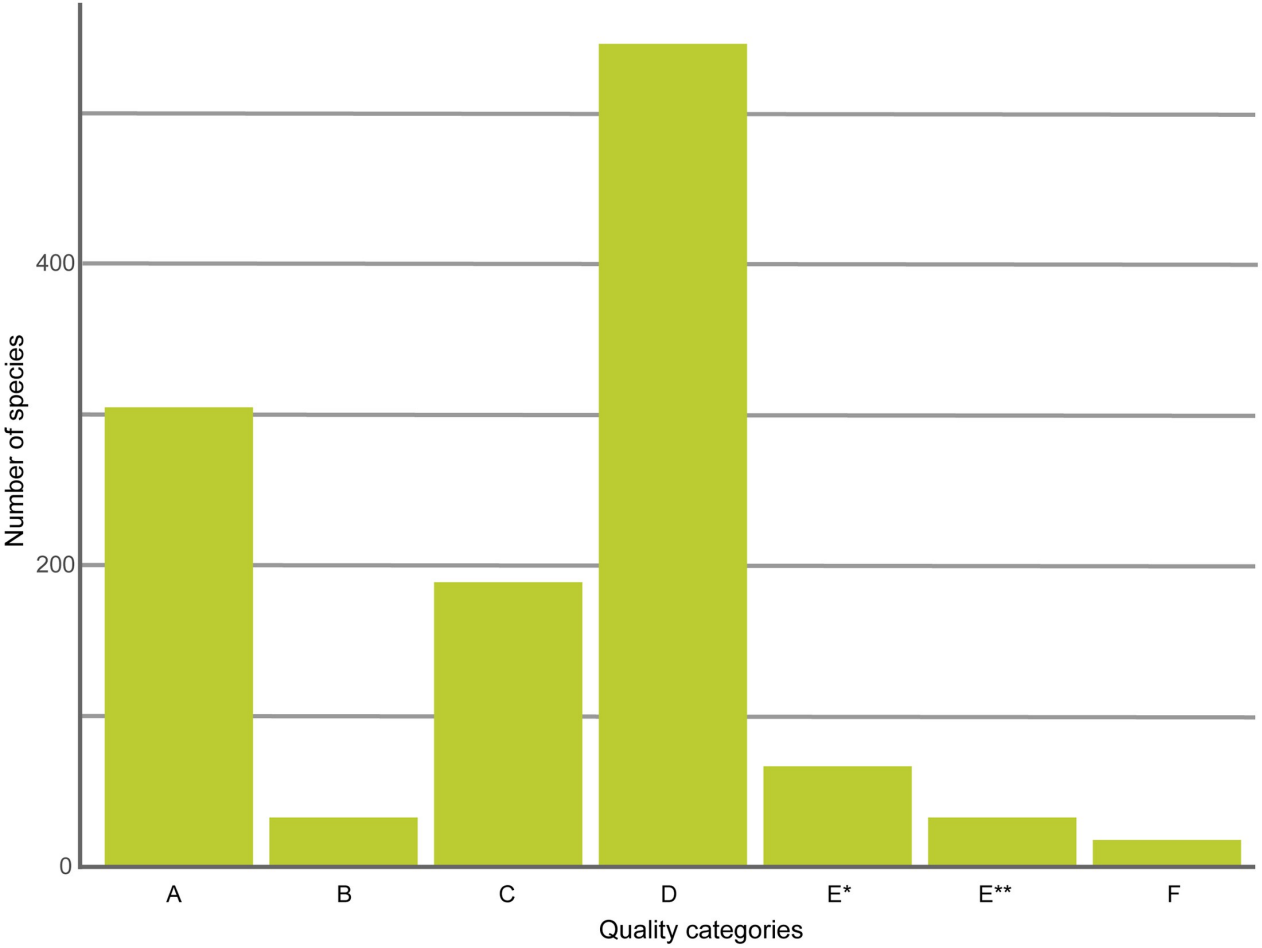
Composition of BIN-based classification (see methods for more details) for Peruvian Amazonian bird species based on records in BOLD.

Quality analysis showed us that 28.38% (338 species) of the Peruvian Amazonian species registered in BOLD have good representation (categories A/B) (Fig 6). There is also a significant percentage of species (45.84%, 546 species) that are currently data deficient (Fig 6).

## Discussion

DNA-based biomonitoring methods, including those using metabarcoding, have many advantages [37, 38], especially for areas difficult to access such as the Amazonian region in Peru [15]. However, current limitations for the proper employment of these methods include the lack of completeness of publicly accessible reference libraries [37].

We created an updated list of Peruvian Amazonian birds incorporating recent curated records from a citizen science platform such as eBird (ebird.org), corroborated with literature search. Our results highlight the importance of the conservation of the Amazon rainforest in Peru, as it is home to 79.5% of Peru’s bird species and approximately 13.7% of the world’s bird species.

Using this comprehensive species list, our gap analysis revealed that 87.4% of species had at least one of the four markers screened. However, this estimate decreases to 79.2% if we only include public sequences and decreases drastically to 4.3% when only sequences obtained from birds sampled in Peru are considered. Furthermore, the fact that 43.8% of Peruvian Amazonian endemic species (n = 32) have no sequences of the analyzed markers highlights the challenge of detecting and sampling species that have highly restricted distributions and small populations (Table 1).

This lack of local coverage means the current genetic information available in public reference libraries can fail to capture intraspecific variation, local subspecies that can result from structured populations, and cryptic species. Although many bird species are considered to be widely distributed throughout the Neotropical region, recent studies on variation in genetic structure have shown that there are independent evolutionary units within these species, and these groups have been taxonomically reevaluated [39].

This is more concerning given that we found species that are only represented by samples taken from outside the Amazon Basin, and even outside of the Neotropical zoogeographical realm. The difference in the number of sequences uploaded from samples collected in Peru in comparison to other countries in South America can be attributed to several factors. These include a limited local infrastructure compared to other countries, limited local investment in life sciences, a lack of experts, and delays in research permits.

Another aspect worthy of consideration is the number of data deficient (D) species in these reference libraries found by our quality analysis. We recommend that future efforts should aim to increase the number of records that include not only sequences but also detailed information of the source of the sample, the museum voucher, and georeferenced data. This is important because taxonomic validation of museum specimens can help avoid incorrect species identification [40].

Results also revealed that there are currently 24.27% (289 species) of records that point to possible cryptic diversity or BIN incongruences (classified as C, E* or E** in the quality analysis) (Fig 6, S1 File). These groups should be further studied in future phylogenetics projects as BIN incongruence has shown to be an effective way to signal hidden diversity [36].

Considering that national species lists are continuously being updated, and that, in Peru in particular, there are several species currently in a hypothetic category with records near the border with Ecuador, Colombia, Brazil and Chile [7], it would be important to think more regionally within barcoding and molecular reference libraries campaigns, to coordinate among countries that share regions in the Amazonia. Our analysis also highlights the need for up-to-date species lists, that consider current information about recent geographic distribution reports, newly described species, and current nomenclature. This is crucial for assessing the strengths and deficiencies of reference libraries. Without in-depth analysis, information gaps can be missed because major databases may not update their nomenclature regularly enough [41].

The current gap-analysis, considering the length of the sequences of the four markers analyzed, can provide a good picture of the amount of genetic information available for eDNA and metabarcoding studies. This is because for these studies even the shortest sequence found (113 base pairs) can be used to determine the taxonomic identity of a sample [14, 16]. However, other lines of research such as phylogenetics might need longer sequences.

We recommend that to fill the current information gap the establishment of independent facilities inside the country should be promoted, so that the complete workflow from sample collecting to DNA sequencing can be done without the need for sending the samples outside the country [42]. This brings the benefits of improving the local scientist education in lab practices [42].

In recent decades improved protocols for obtaining DNA and sequencing technology have expanded the potential of natural history collections, as the number of specimens useful for molecular studies has now increased [43, 44]. Considering that many of the Peruvian Amazonian bird species that are not currently represented in public reference databases are endemic species with restricted distribution ranges (which can increase sampling difficulty) we propose the use of scientific collections as a museomic resource in the creation of molecular libraries to accelerate and facilitate the procurement of DNA.

In conclusion, our results show Peruvian Amazonian birds are well represented at a global scale, but poorly represented at local level with few records from Peru. This is reflected in the low coverage of endemic species in comparison with widespread species and in the BIN incongruences identified which probably indicate hidden diversity. Our results help to identify underrepresented groups and areas in order to focus futures studies on bird biodiversity. Mechanisms to improve local coverage should include strengthening local genomics training and facilities, as well the use of scientific collections from natural history museums as a source of genetic information.

## Supporting information

S1 File Database of Peruvian bird species and their genetic representation. (XLSX)

S1 Table Number of species of Amazonian birds, number of species that are represented by at least one of the four markers analyzed in GenBank or BOLD, and the percentage of representation per taxonomic order. (PDF)
