# Supplementary material for: Lack of local genetic representation in one of the regions with the highest bird species richness, the Peruvian Amazonia
Source: PLoS One. 2024 Jan 2;19(1):e0296305. doi: 10.1371/journal.pone.0296305 (PMC10760656; doi:10.1371/journal.pone.0296305)
Supplement: S1 Table — (PDF) [file pone.0296305.s002.pdf]

S2 Table. Number of species of Amazonian birds, number of species that are represented by at least one of the four markers analyzed in GenBank or BOLD, and the percentage of representation per taxonomic order.

Order	Species	Species with public seqs	Species with public seqs (%)	Species with seqs	Species with seqs (%)
Accipitriformes	49	48	97.96	48	97.96
Anseriformes	19	19	100.00	19	100.00
Apodiformes	117	86	73.50	92	78.63
Caprimulgiformes	25	20	80.00	21	84.00
Charadriiformes	39	39	100.00	39	100.00
Ciconiiformes	3	3	100.00	3	100.00
Columbiformes	22	21	95.45	22	100.00
Coraciiformes	9	8	88.89	9	100.00
Cuculiformes	15	13	86.67	14	93.33
Eurypygiformes	1	1	100.00	1	100.00
Falconiformes	17	16	94.12	17	100.00
Galliformes	15	14	93.33	14	93.33
Gruiformes	22	19	86.36	19	86.36
Opisthocomiformes	1	1	100.00	1	100.00
Passeriformes	926	697	75.27	791	85.42
Pelecaniformes	23	22	95.65	22	95.65
Piciformes	90	70	77.78	80	88.89
Podicipediformes	4	4	100.00	4	100.00
Psittaciformes	45	43	95.56	44	97.78
Strigiformes	29	22	75.86	25	86.21
Suliformes	2	2	100.00	2	100.00
Tinamiformes	22	15	68.18	19	86.36
Trogoniformes	11	10	90.91	10	90.91
